# Risk Factors Associated With Hospital Readmission and Costs for Pouchitis

**DOI:** 10.1093/crocol/otab006

**Published:** 2021-03-27

**Authors:** Adalberto Gonzalez, Kapil Gupta, Asad Ur Rahman, Vaibhav Wadhwa, Bo Shen

**Affiliations:** 1 Department of Gastroenterology and Hepatology, Cleveland Clinic Florida, Weston, Florida, USA; 2 Department of Gastroenterology and Hepatology, Rutgers Robert Wood Johnson Medical School, New Brunswick, New Jersey, USA; 3 Center for Advanced Endoscopy, Beth Israel Deaconess Medical Center/Harvard Medical School, Boston, Massachusetts, USA; 4 Center for Inflammatory Bowel Disease, Columbia University Irving Medical Center-NewYork Presbyterian Hospital, New York, New York, USA

**Keywords:** ileal pouch-anal anastomosis, inflammatory bowel disease, pouchitis, readmission

## Abstract

**Background:**

Pouchitis is the most common long-term complication in patients with restorative proctocolectomy and ileal pouch-anal anastomosis. This study aimed to identify readmission rates for pouchitis and risk factors associated with readmissions in an extensive national database.

**Methods:**

We performed a retrospective analysis using the National Readmission Database to determine if patient demographics and clinical characteristics were predictors of hospital readmission within 30 days for adult patients (age >18 years) discharged with a principal diagnosis of pouchitis (ICD-9 code—569.71) from January 2013 to December 2013. Both univariable and multivariable analyses were performed to assess factors associated with 30-day readmission.

**Results:**

A total of 1538 patients with pouchitis who were discharged alive were identified. 10.2% [95% confidence interval: 7.6, 12.7] of these were readmitted within 30 days of discharge. The average days to readmission were 18.6 ± 1.01. Multivariable analysis of risk factors associated with readmission showed older age as a protective factor for readmission [odds ratio (OR) = 0.88 (0.81, 0.96); *P* < 0.005]. Sex and the presence of permanent ileostomy were not associated with readmission in patients with pouchitis. The length of stay during readmissions was associated with postoperative wound infection [OR = 7.7 (94.0, 11.30); *P* < 0.001], ileus [OR = 4.5 (1.6, 7.4); *P* < 0.002], permanent ileostomy [OR = 3.7 (1.7, 5.7); *P* < 0.001], and long-term use of nonsteroidal anti-inflammatory drugs [OR = 3.2 (1.06, 5.3); *P* < 0.003].

**Conclusions:**

Readmissions in pouchitis patients are frequent. Long-term use of nonsteroidal anti-inflammatory drugs, ileus, permanent ileostomy, and postoperative wound infection is associated with increased length of stay in readmissions.

## INTRODUCTION

Pouchitis is an inflammatory condition of the ileal pouch reservoir of an ileal pouch-anal anastomosis (IPAA).^1^ It is the most frequently observed long-term complication of IPAA, occurring in about 23%–59% of patients who undergo this procedure.^2–5^ The pathogenesis of pouchitis is unclear, but it is thought to result from an abnormal immune response to altered luminal and mucosal bacteria in genetically susceptible hosts. It represents a disease spectrum ranging from acute antibiotic-responsive pouchitis to chronic antibiotic-refractory pouchitis.^1^

Reported risk factors for pouchitis include a history of ulcerative colitis (UC),^6^ primary sclerosing cholangitis,^7^ the use of nonsteroidal anti-inflammatory drugs (NSAIDs),^8^ smoking history,^9^ multiple prior abdominal surgeries,^10^ and immune-mediated disorders.^11^ Patients with severe pouchitis may require hospitalization due to abdominal pain, diarrhea, dehydration, and failure to thrive. Wu et al found that male sex^12^ is a risk factor for pouchitis while female sex has been cited by another study.^13^ There are a few studies on the readmission rate in UC.^14,15^ To date, there are no long-term data on hospitalization or readmission rates for pouchitis. A 2009 retrospective study by Datta et al^16^ investigated hospital readmission rates after IPAA surgery and reported a 30% readmission rate over 5 years. To our knowledge, there have been no previous studies observing hospital readmission after the initial hospitalization for the primary diagnosis of pouchitis. We aimed to determine the rate and risk factors of readmission within 30 days after admission for pouchitis.

## MATERIALS AND METHODS

We used the National Readmission Database (NRD) to perform a retrospective analysis. NRD is part of the Healthcare Cost and Utilization Project (HCUP) of the Agency for Healthcare Research and Quality. NRD is the largest publicly available database that contains deidentified information of inpatient records from most hospitals in participating states. It is derived from the State Inpatient Databases (SID) that contains specific patient linkage numbers which can be used to track a person across hospitals within a particular state. NRD accounts for approximately 51.2% of the US population and 49.3% of all hospitalizations, making it a good representation of total US population. After applying appropriate weights, NRD contains data from roughly 35 million discharges in the United States.^17^

### Inclusion and Exclusion Criteria

We queried the NRD using ICD-9 diagnosis code for pouchitis (ICD-9 code—569.71) in the primary diagnosis field to extract our study population. We evaluated demographic and clinical factors for hospital readmission within 30 days for adult patients (age >18 years) discharged with a principal diagnosis of pouchitis from January 2013 to December 2013. Index stays were identified from January to November 2013 to allow for a 30-day readmission window. Index stays required “live” discharge status and nonmissing length of stay (LOS). Clinical variables were based on secondary diagnoses at the time of the first admission. These included patients with prior history of UC, Crohn disease (CD), and familial adenomatous polyposis. We excluded patients without a principal diagnosis of pouchitis.

All ICD-9 codes used for classifications can be found in Appendix 1.

### Demographic and Clinical Variables

Of the included variables, median household income and primary expected payer had missing values (1.6% and 0.3%, respectively), and these observations were dropped from the final multivariable analysis if said variables were included.

### Outcome Measures

The primary outcome of our study was to assess 30-day readmission in patients admitted with pouchitis. The secondary aims of the study were to calculate factors associated with 30-day readmission, LOS in the hospital, and costs incurred during hospitalization.

### Statistical Analysis

Univariable analysis was performed to assess differences between subjects with and without 30-day readmission; continuous variables were compared using *t* tests, and categorical variables were compared using Rao–Scott chi-square tests. Also, multivariable logistic regression analysis was performed to assess factors associated with 30-day readmissions. Linear regression analysis was also executed to evaluate factors associated with LOS and costs. All multivariable models were chosen using backward elimination variable selection; sex and number of comorbidities were included in all readmission models, and the elimination process continued until other variables in the model had a *P* < 0.10. A similar selection method was used to choose the final models for LOS and costs; only the sex was forced included in this case. All analyses were performed using SAS Survey Procedures (version 9.4, The SAS Institute, Cary, NC), and a *P* < 0.05 was considered statistically significant.

## RESULTS

A total of 1538 subjects with pouchitis who were discharged alive were identified (Table 1). One hundred fifty-six patients [10.2%; 95% confidence interval (CI): 7.6, 12.7] of these were readmitted within 30 days of discharge. We identified 629 patients who died with a diagnosis of pouchitis, and only 1 of these died in the hospital. Because we could not assess readmission risk if they died while in the hospital, we have excluded this patient. Population weighted in-hospital mortality was 0.14%.

**Table 1.** Patient and Hospital Characteristics

Factor	Total (N = 1538)	No 30-Day Readmission (N = 1381)	30-Day Readmission (N = 156)	*P*
Age (years), mean ± SE	46.6 ± 0.78	47.1 ± 0.81	42.0 ± 2.0	0.015
Age				0.13
18–64 years	218 (14.2)	205 (14.8)	13 (8.5)	
65+ years	1319 (85.8)	1176 (85.2)	143 (91.5)	
Sex				0.9
Male	715 (46.5)	641 (46.4)	74 (47.3)	
Female	823 (53.5)	740 (53.6)	82 (52.7)	
Primary expected payer				0.17
Medicare	343 (22.4)	315 (22.8)	29 (18.2)	
Medicaid	88 (5.7)	77 (5.6)	10 (6.7)	
Private insurance	934 (60.9)	847 (61.5)	88 (56.1)	
Other	168 (11.0)	139 (10.1)	30 (19.0)	
Median household income				0.77
$1–37,999	247 (16.4)	217 (16.0)	29 (19.5)	
$38,000–47,999	411 (27.2)	365 (26.9)	45 (30.0)	
$48,000–63,999	403 (26.7)	371 (27.3)	32 (21.0)	
$64,000+	448 (29.7)	404 (29.7)	45 (29.5)	
UC	512 (33.3)	448 (32.4)	64 (41.2)	0.18
CD	282 (18.3)	248 (18.0)	33 (21.2)	0.55
IBD (CD or UC)	781 (50.8)	683 (49.4)	98 (62.5)	0.065
Familial adenomatous polyposis	21 (1.3)	18 (1.3)	2 (1.4)	0.98
*Clostridium difficile*	60 (3.9)	56 (4.0)	5 (3.1)	0.68
Ileus	99 (6.4)	88 (6.4)	10 (6.7)	0.95
Postoperative wound infection	26 (1.7)	26 (1.9)	0 (0)	—
*H. pylori*	0 (0)	0 (0)	0 (0)	—
Peptic ulcer disease	30 (1.9)	24 (1.7)	6 (3.9)	0.23
CMV	4 (0.25)	4 (0.28)	0 (0)	—
Intestinal/colonic ischemia	12 (0.76)	12 (0.85)	0 (0)	—
Diabetes type I	5 (0.32)	4 (0.25)	1 (0.95)	0.32
Long-term use of NSAIDs	4 (0.25)	4 (0.27)	0 (0)	—
Autoimmune disease	217 (14.1)	193 (14.0)	24 (15.5)	0.77
Autoimmune disease (unspecified)	0 (0)	0 (0)	0 (0)	—
Celiac disease	6 (0.42)	3 (0.24)	3 (2.0)	0.042
PSC	60 (3.9)	51 (3.7)	9 (5.7)	0.37
Asthma	91 (5.9)	84 (6.1)	6 (4.1)	0.49
RA	24 (1.6)	24 (1.7)	0 (0)	—
Graves disease	7 (0.42)	3 (0.24)	3 (2.0)	0.033
Hashimotos thyroiditis	3 (0.22)	3 (0.25)	0 (0)	—
Psoriasis	24 (1.6)	24 (1.7)	0 (0)	—
SLE	3 (0.20)	3 (0.23)	0 (0)	—
AIHA	3 (0.17)	0 (0)	3 (1.7)	—
Vitiligo	1 (0.09)	1 (0.10)	0 (0)	—
Pernicious anemia	0 (0)	0 (0)	0 (0)	—
Idiopathic thrombocytopenic purpura	5 (0.32)	5 (0.36)	0 (0)	—
Multiple sclerosis	10 (0.65)	10 (0.72)	0 (0)	—
Venous thrombosis	21 (1.4)	19 (1.4)	2 (1.4)	0.97
Dehydration	267 (17.3)	233 (16.8)	34 (21.9)	0.3
Diarrhea	308 (20.1)	269 (19.5)	39 (25.1)	0.3
OSA	10 (0.64)	10 (0.71)	0 (0)	—
Obesity	87 (5.6)	77 (5.6)	10 (6.1)	0.83
NAFLD	18 (1.1)	16 (1.2)	2 (0.99)	0.88
Metabolic syndrome	0 (0)	0 (0)	0 (0)	—
COPD	37 (2.4)	35 (2.6)	2 (1.3)	0.5
No. comorbidities				0.37
0	401 (26.1)	359 (26.0)	43 (27.3)	
1	454 (29.6)	413 (29.9)	42 (26.8)	
2	334 (21.7)	309 (22.4)	25 (15.8)	
3+	348 (22.6)	301 (21.8)	47 (30.1)	
Bed size of hospital				0.47
Small	186 (12.1)	171 (12.4)	15 (9.5)	
Medium	276 (18.0)	255 (18.5)	21 (13.5)	
Large	1076 (69.9)	955 (69.1)	120 (77.0)	
Permanent ileostomy	55 (3.6)	44 (3.2)	11 (7.0)	0.18
LOS (days), mean ± SE	5.6 ± 0.35	5.7 ± 0.39	4.8 ± 0.41	0.093
Total charges ($), mean ± SE	42,197.5 ± 3438.1	42,380.0 ± 3752.7	40,584.8 ± 4546.5	0.75

Data presented as weighted frequency (%) unless otherwise stated.

AIHA, autoimmune hemolytic anemia; CMV, cytomegalovirus; COPD, chronic obstructive pulmonary disease; NAFLD, non-alcoholic fatty liver disease; OSA, obstructive sleep apnea; PSC, primary sclerosing cholangitis; RA, rheumatoid arthritis; SLE, systemic lupus erythematosus.

### Patient Characteristics

Patients in the 30-day readmission group were, on average, younger than patients in the nonreadmission group (42.0 vs 47.1; *P* = 0.015). We found no significant difference in sex, type of healthcare insurance, or median household income. The 30-day readmission group was more likely to be associated with UC than with CD or familial adenomatous polyposis, although this was not statistically significant (*P* = 0.18). The LOS was lower in the readmission group compared to the nonreadmission group (4.8 vs 5.7, respectively), but this was not statistically significant (*P* = 0.09). Similarly, there was no significant difference in total charges between both groups.

### Factors Associated With 30-Day Readmission

The average duration to readmission was 18.6 ± 1.01 days. The initial evaluation showed that younger age, and the presence of celiac disease or Graves disease were associated with 30-day readmission. Multivariable analysis of risk factors associated with 30-day readmission (Table 2) showed older age as a protective factor for readmissions [odds ratio (OR) = 0.88 (95% CI: 0.81, 0.96); *P* < 0.005]. There was no difference in readmission rates for either sex. Patients with 3 or more comorbidities and those with permanent ileostomy were more likely to be readmitted, but these were not statistically significant (*P* = 0.13 and *P* = 0.11, respectively). A patient’s diagnosis of celiac disease was more likely to be associated with readmission [OR = 6.5 (95% CI: 1.06, 39.4); *P* = 0.043].

**Table 2.** Multivariable Analysis of Factors Associated With 30-Day Readmission

Factor	OR (95% CI)	*P*
Age (years)	0.88 (0.81, 0.96)	0.005
Female vs male	0.89 (0.49, 1.6)	0.69
No. comorbidities		
1 vs 0	0.93 (0.46, 1.9)	0.85
2 vs 0	0.82 (0.35, 1.9)	0.65
3+ vs 0	1.8 (0.84, 3.9)	0.13
Celiac disease	6.5 (1.06, 39.4)	0.043
Permanent ileostomy	2.9 (0.80, 10.5)	0.11

### Factors Associated With LOS

The risk factors associated with LOS during readmission were analyzed (Table 3). Prolonged LOS in patients who were subsequently readmitted for pouchitis was associated with postoperative wound infection [OR = 7.7 (95% CI: 94.0, 11.30); *P* < 0.001], ileus [OR = 4.5 (95% CI: 1.6, 7.4); *P* < 0.002], permanent ileostomy [OR = 3.7 (95% CI: 1.7, 5.7); *P* < 0.001], or long-term use of NSAIDs [OR = 3.2 (95% CI: 1.06, 5.3); *P* < 0.003]. Patients with 2 or more comorbidities also had longer LOS [OR = 2.3 (95% CI: 1.2, 3.4); *P* < 0.001]. Venous thrombosis was associated with a LOS of 12.5 longer, but this was not significant as the values have a wide range (95% CI: −0.18, 25.2; *P* = 0.053). *Clostridium difficile* infection was not associated with LOS (95% CI: −1.6, 3.9; *P* = 0.42).

**Table 3.** Analysis of Factors Associated With LOS

	Unadjusted Analysis		Multivariable Analysis	
Factor	Parameter (95% CI)	*P*	Parameter (95% CI)	*P*
65+ vs 18–64 years old	0.29 (−0.93, 1.5)	0.64	1.8 (−0.08, 3.7)	0.06
Female vs male	0.10 (−1.2, 1.4)	0.88	—	—
Primary expected payer				
Medicaid vs Medicare	2.4 (−6.7, 11.6)	0.6	—	—
Private insurance vs Medicare	−1.4 (−2.8, 0.10)	0.068	—	—
Other vs Medicare	−1.5 (−3.3, 0.24)	0.09	—	—
Median household income				
$38,000–47,999 vs $1–37,999	−1.2 (−2.5, 0.03)	0.056	—	—
$48,000–63,999 vs $1–37,999	−0.41 (−2.7, 1.9)	0.73	—	—
$64,000+ vs $1–37,999	−0.83 (−2.3, 0.63)	0.26	—	—
UC	0.13 (−1.6, 1.9)	0.88	—	—
CD	1.03 (−0.32, 2.4)	0.13	—	—
Familial adenomatous polyposis	0.85 (−3.4, 5.1)	0.69	—	—
*Clostridium difficile*	1.2 (−1.6, 3.9)	0.42	—	—
Ileus	5.1 (2.2, 8.1)	<0.001	4.5 (1.6, 7.4)	0.002
Postoperative wound infection	8.2 (4.0, 12.4)	<0.001	7.7 (4.0, 11.3)	<0.001
Peptic ulcer disease	−1.00 (−2.8, 0.75)	0.26	—	—
CMV	2.8 (−3.1, 8.7)	0.35	—	—
Intestinal/colonic ischemia	−0.34 (−2.7, 2.0)	0.77	—	—
Diabetes type I	−1.9 (−2.8, −0.99)	<0.001	—	—
Long-term use of NSAIDs	3.4 (−0.87, 7.6)	0.12	3.2 (1.06, 5.3)	0.003
Autoimmune disease	−0.24 (−1.9, 1.4)	0.78	—	—
Venous thrombosis	13.1 (0.10, 26.1)	0.048	12.5 (−0.18, 25.2)	0.053
Dehydration	−1.01 (−2.2, 0.15)	0.087	—	—
Diarrhea	−0.12 (−1.4, 1.1)	0.86	—	—
OSA	−3.1 (−4.2, −2.0)	<0.001	−2.7 (−4.5, −0.89)	0.003
Obesity	7.5 (−2.3, 17.4)	0.13	—	—
NAFLD	−0.43 (−4.1, 3.2)	0.82	—	—
COPD	0.38 (−2.5, 3.3)	0.79	—	—
No. comorbidities				
1 vs 0	0.47 (−0.38, 1.3)	0.28	0.50 (−0.27, 1.3)	0.2
2 vs 0	2.1 (0.95, 3.2)	<0.001	2.3 (1.2, 3.4)	<0.001
3+ vs 0	3.7 (1.06, 6.4)	0.006	3.9 (0.91, 6.9)	0.011
Bed size of hospital				
Medium vs small	−3.4 (−8.0, 1.2)	0.15	—	—
Large vs small	−2.4 (−6.9, 2.2)	0.31	—	—
Permanent ileostomy	4.3 (1.3, 7.2)	0.004	3.7 (1.7, 5.7)	<0.001

CMV, cytomegalovirus; COPD, chronic obstructive pulmonary disease; NAFLD, non-alcoholic fatty liver disease; OSA, obstructive sleep apnea.

### Analysis of Factors Associated With Costs

Increased costs were also significantly associated with certain risk factors (Table 4). These included postoperative wound infection [$72,051 (95% CI: 21,922, 122,179); *P* = 0.005], long-term use of NSAIDs [$97,454 (95% CI: 57,607, 137,300)]; *P* < 0.001), 2 or more comorbidities [$11,917 (95% CI: 1737, 22,096); *P* = 0.11], and permanent ileostomy [$30,533 (95% CI: 6074, 54,992); *P* = 0.014]. Ileus was associated with an increased cost of $68,475 but this was not statistically significant (95% CI: −12,438, 149,389; *P* = 0.97). Also, venous thrombosis conferred an increased cost of $53,423 that was not statistically significant (95% CI: −4798, 111,644; *P* = 0.072).

**Table 4.** Analysis of Factors Associated With Costs

	Unadjusted Analysis		Multivariable Analysis	
Factor	Parameter (95% CI)	*P*	Parameter (95% CI)	*P*
65+ vs 18–64 years old	−1360 (−12,405, 9684)	0.81	—	—
Female vs male	−11,733 (−24,549, 1082)	0.073	—	—
Primary expected payer				
Medicaid vs Medicare	−18,346 (−46,019, 9327)	0.19	—	—
Private Insurance vs Medicare	−13,629 (−38,609, 11,351)	0.28	—	—
Other vs Medicare	−14,576 (−40,948, 11,796)	0.28	—	—
Median household income				
$38,000–47,999 vs $1–37,999	−12,583 (−25,155, −11)	0.05	—	—
$48,000–63,999 vs $1–37,999	−11,250 (−22,630, 131)	0.053	—	—
$64,000+ vs $1–37,999	715 (−21,718, 23,147)	0.95	—	—
UC	−6485 (−17,200, 4231)	0.24	—	—
CD	4327 (−7228, 15,883)	0.46	—	—
Familial adenomatous polyposis	14,972 (−29,414, 59,358)	0.51	—	—
*Clostridium difficile*	−4380 (−22,090, 13,331)	0.63	—	—
Ileus	74,191 (−7234, 155,617)	0.074	68,475 (−12,438, 149,389)	0.097
Postoperative wound infection	79,311 (25,494, 133,129)	0.004	72,051 (21,922, 122,179)	0.005
Peptic ulcer disease	6308 (−18,822, 31,439)	0.62	—	—
CMV	−4693 (−45,967, 36,581)	0.82	—	—
Intestinal/colonic ischemia	−1521 (−22,880, 19,837)	0.89	—	—
Diabetes type I	−22,677 (−33,057, −12,297)	<0.001	−27,468 (−55,428, 491)	0.054
Long-term use of NSAIDs	101,512 (49,547, 153,478)	<0.001	97,454 (57,607, 137,300)	<0.001
Autoimmune disease	18,767 (−19,583, 57,116)	0.34	—	—
Venous thrombosis	61,178 (5261, 117,095)	0.032	53,423 (−4798, 111,644)	0.072
Dehydration	−7797 (−18,826, 3233)	0.17	—	—
Diarrhea	−4730 (−15,636, 6176)	0.4	—	—
OSA	−27,422 (−34,971, −19,873)	<0.001	−28,697 (−45,108, −12,285)	<0.001
Obesity	11,378 (−9034, 31,790)	0.27	—	—
NAFLD	−6056 (−28,987, 16,875)	0.6	−19,294 (−43,894, 5306)	0.12
COPD	27,329 (−12,491, 67,149)	0.18	—	—
No. comorbidities				
1 vs 0	2400 (−4969, 9769)	0.52	2184 (−4937, 9305)	0.55
2 vs 0	12,890 (1772, 24,009)	0.023	11,917 (1737, 22,096)	0.022
3+ vs 0	32,225 (6923, 57,526)	0.013	30,840 (6946, 54,734)	0.011
Bed size of hospital				
Medium vs small	−1892 (−16,621, 12,837)	0.8	—	—
Large vs small	9119 (−6460, 24,698)	0.25	—	—
Permanent ileostomy	40,272 (17,487, 63,057)	<0.001	30,533 (6074, 54,992)	0.014

CMV, cytomegalovirus; COPD, chronic obstructive pulmonary disease; NAFLD, non-alcoholic fatty liver disease; OSA, obstructive sleep apnea.

## DISCUSSION

Our study, based on a nationally representative US readmission database, found that patients admitted for pouchitis had a 10.2% 30-day readmission rate. The average days to readmission were 18.6 ± 1.01. In multivariable analysis of risk factors, older age was a protective factor for readmissions with younger age being a risk factor. Interestingly, presence of celiac disease increased the risk of readmission. Increased LOS in readmissions was associated with postoperative wound infection, ileus, permanent ileostomy, or a long-term use of NSAIDs. This study is unique as there are no previous studies examining risk factors for readmission in pouchitis to our knowledge.

There are several previous studies presenting readmission data for UC. A 2017 retrospective study using the US NRD found a 10.6% readmission rate for UC, while another abstract found that 19% of inflammatory bowel disease (IBD)-related hospitalizations were readmitted within 30 days.^15^ Of note, Datta et al reported a 30% readmission rate after IPAA creation.^16^ Patients with UC or CD comprised the majority (41.2% and 21.3%, respectively) of our readmission group, which may have led to the similarity of pouchitis readmission rate in our study (10%) to 30-day readmission rates for UC^14^ (10.6%) and IBD^15^ (19%) in previous studies. The presence of underlying UC is the most critical risk factor for pouchitis, so it is no surprise that IBD composed a majority of our patient population. In turn, pouchitis occurs less commonly in CD and rarely occurs in familial adenomatous polyposis,^1^ as was seen in our study. This supports the theory that the pathogenetic background of UC may contribute significantly to the development of pouchitis.^18^

In our study, the average number of days for readmission for pouchitis was 18.6 ± 1.01 days. A prospective cohort study of patients with prior history of UC showed that a longer follow-up period was associated with chronic pouchitis,^6^ but it did not show data on 30-day readmission data. Providing closer outpatient follow-up after hospital discharge may likely limit the number of readmissions for pouchitis. Close outpatient follow-up has previously been postulated as a way to reduce readmissions for UC.^14^ Follow-up within 2 weeks may be ideal since the average time to readmissions was 18 days.

We observed that patients who were readmitted within 30 days had a lower mean age compared to patients who did not get readmitted. There was however no difference seen in patients above or below the age of 65 years. However, it also leads to an average increase of 1.8 days in LOS. The prolonged LOS is likely secondary to an increase in comorbidities, which was also associated with an increase in LOS by 2.1 days. Although there are no specific data observing association of age and readmission in pouchitis, some previous studies have shown that younger age may be associated with higher rates of chronic pouchitis and UC flares. A retrospective study, which included 184 patients, showed that young age at proctocolectomy is associated with chronic pouchitis.^19^ Shorter disease duration may also be a risk factor for chronic pouchitis.^6^ Of note, UC readmission rates have been associated with younger age and male sex.^14^ A Canadian retrospective study in readmissions also found that older patients had fewer readmissions but a 40-fold increase in mortality, which may contribute to fewer readmissions in the elderly.^20^ We did not record data on the age of pouchitis patients excluded due to mortality to compare it to the study by Nguyen et al.^20^ The trend between age and increased readmission rates may be generalized to other inpatient diagnoses as well. A retrospective national database study found that 30-day readmission rates for all conditions steadily decreased after the age of 45 and significantly decreased after the age of 65.^21^ Consistent with previous conflicting data regarding either sex as a risk factor for pouchitis,^12,13^ neither sex was associated with an increased risk of readmission, LOS, or healthcare costs for pouchitis in our study.

In our multivariate analysis, celiac disease was found to be associated with an increase in 30-day readmission. Celiac disease has been postulated as a secondary cause of refractory pouchitis, but no clear risk has been established.^1^ Our findings are likely the result of frequent testing for celiac disease in patients with chronic antibiotic-resistant pouchitis and an apparent increased rate of false-positive celiac serology in patients with IPAA. A 2010 retrospective study, including 126 patients at the Cleveland Clinic Pouchitis Clinic, found that false-positive results for celiac serology were common (47%) in patients with IPAA and chronic antibiotic-refractory pouchitis.^22^ And although genetic and immunologic overlap exists between IBD and celiac disease, no apparent risk has been associated either.^23^ Primary sclerosing cholangitis^7^ and other autoimmune diseases^11^ have been linked to an increased risk for pouchitis. Our study found no increased risk in regards to readmission rate, LOS, or hospital costs with primary sclerosing cholangitis or any other autoimmune diseases besides celiac disease. Since only 6 patients had a diagnosis of celiac disease, larger studies are needed to verify this association found by our study.

Gastrointestinal infections, such as cytomegalovirus^24^ and *C. difficile*,^25^ are also risk factors for pouchitis. However, our data showed similar rates of *C. difficile* infection and cytomegalovirus infections among pouchitis patients who were readmitted compared to those who were not readmitted.

Postoperative wound infection and ileus significantly increased LOS of readmission (7.7 and 4.5 days, respectively). Although we were unable to specify the timing of readmission, these likely mainly represent 2 postoperative complications after IPAA creation. A retrospective study looking at hospital readmission rates after IPAA found a 30% readmission rate and more than the doubled LOS; the 2 most common causes were small bowel obstruction and sepsis/anastomotic leak.^16^ Our data support the fact that mechanical or functional obstruction, or infection can lead to readmission in pouchitis.

The use of NSAIDs was associated with longer LOS by an average of 3.2 days in our study. NSAID use is a known risk factor for pouchitis.^26^ A retrospective case–control study involving 80 patients showed that NSAID use was a risk factor for both acute and chronic pouchitis.^8^ However, no previous studies have investigated NSAID use as a risk factor for readmission LOS or costs. This increased LOS further supports the belief that NSAID use should be discontinued in patients with IPAA. Since most NSAIDs are available over the counter, these findings strengthen our recommendations to our patients and primary care providers to help decrease LOS and costs incurred during hospitalization.

A case–control study demonstrated that pouchitis is a significant driver of healthcare costs among patients with a prior history of chronic UC.^27^ Furthermore, a systematic review studying the financial burdens of postsurgical complications with UC found that pouchitis, pouch failure, and small bowel obstruction were associated with the highest cost.^28^ We found increased costs with many of the factors that increased LOS in readmissions. These factors included postoperative wound infections, permanent ileostomy, long-term use of NSAIDs, and the presence of 2 or more comorbidities. The presence of ileus is numerically associated with greater hospital costs. It seems that postsurgical complications of IPAA are a substantial contributor to increased LOS and healthcare costs in pouchitis.

Our study has several limitations. First, since we used a national database, the data rely on administrative health claims, so the coded diagnoses could not be directly verified. Additionally, the severity of pouchitis, the different subtypes of pouchitis, and the associated acuity and severity of IBD could not be determined for the admissions. Third, we were unable to evaluate the timing of the readmission in relation to IPAA creation, which may have added valuable information. Finally, it was not feasible to study outpatient office visits in between the initial hospitalizations and readmission. This is similar to other national database studies.^14^

## CONCLUSIONS

In summary, we found that 10.2% of patients with pouchitis required readmission within 30 days, usually at around 18 days after discharge. The presence of long-term use of NSAIDs, ileus, permanent ileostomy, and postoperative wound infection is associated with an increased LOS and increased healthcare costs from readmissions. These risk factors can be used to identify pouchitis patients with a higher risk for readmission. These patients should be more closely monitored to avoid readmission and reduce healthcare costs. Future studies are needed to further understand approaches to decrease readmissions in pouchitis patients.

## Supplementary Material

otab006_suppl_Supplementary_Appendix

## Data Availability

Data are available for review upon reasonable request.

## Notes

Funding: None.
