# Supplementary material for: Risk Factors Associated With Hospital Readmission and Costs for Pouchitis
Source: Crohns Colitis 360. 2021 Mar 27;3(2):otab006. doi: 10.1093/crocol/otab006 (PMC9802153; doi:10.1093/crocol/otab006)
Supplement: otab006_suppl_Supplementary_Appendix [file otab006_suppl_supplementary_appendix.docx]

**Appendix 1. Diagnostic and Procedural ICD-9 Codes**

| ICD-9 Code | Description |
| --- | --- |
| *Diagnostic Codes* |  |
| 569.71 | Pouchitis |
| 556.0-556.9 | Ulcerative Colitis (UC) |
| 555.0-555.9 | Crohn’s Disease (CD) |
| 211.3 | Familial adenomatous polyposis (FAP) |
| 008.45 | Clostridium difficile (CDI) |
| 041.86 | H. pylori |
| 112.85 | Candida |
| 078.5 | CMV |
| 998.59 | Post-operative wound infection |
| 560.1 | Ileus |
| 557.0, 557.1, 557.9 | Intestinal/Colonic Ischemia |
| V58.64 | Long term use of NSAIDs |
| 531.00-531.91, 532.00-532.91. 533.00-533.91, 534.00-534.91 | Peptic ulcer disease |
| 279.49 | Autoimmune diseases (not elsewhere specified) |
| 579.0 | Celiac disease |
| 493.00-493.99 | Asthma |
| 250.01, 250.03, 250.11, 250.13, 250.21, 250.23, 250.31, 250.33, 250.41, 250.43, 250.51, 250.53, 250.61, 250.63, 250.71, 250.73, 250.81, 250.83, 250.91, 250.93 | Diabetes Type I |
| 714.0 | Rheumatoid Arthritis (RA) |
| 242.00-242.01 | Grave’s disease |
| 245.2 | Hashimoto’s thyroiditis |
| 696.0-696.8 | Psoriasis |
| 710.0 | SLE |
| 283.0 | AIAH |
| 709.01 | Vitiligio |
| 281.0 | Pernicious anemia |
| 287.31 | Idiopathic thrombocytopenic purpura |
| 340 | Multiple sclerosis |
| 276.5, 276.50-276.52 | Dehydration |
| 003.0, 003.8-003.9, 004.0-004.3, 004.8-004.9, 008.00-008.04, 008.09  008.1-008.3, 008.41-008.47, 008.49,  008.5, 008.61-008.67, 008.69, 008.8  009.0-009.3, 558.9, 564.5, 787.91 | Diarrhea |
| 278.00, 278.01, 278.03  V85.30-V85-39, V85.41-V85.45 | Obesity |
| 327.23, 780.53, 780.57 | OSA |
| 571.8 | Other chronic nonalcoholic liver disease (NAFLD) |
| 491.0-491.9, 492.0-492.8, 496 | COPD |
| 277.7 | Metabolic syndrome |
| 453.0-453.3, 453.40-453.42, 453.50-453.52, 453.71-453.79, 453.81-453.89, 453.9 | Venous thrombosis |
| *Procedural Codes* |  |
| 46.23 | Permanent Ileostomy |
